# Metronomic Anti-Cancer Therapy: A Multimodal Therapy Governed by the Tumor Microenvironment

**DOI:** 10.3390/cancers13215414

**Published:** 2021-10-28

**Authors:** Raquel Muñoz, Alessandra Girotti, Denise Hileeto, Francisco Javier Arias

**Affiliations:** 1 Department of Biochemistry, Physiology and Molecular Biology, University of Valladolid, Paseo de Belén, 47011 Valladolid, Spain; 2 Smart Biodevices for NanoMed Group, University of Valladolid, LUCIA Building, Paseo de Belén, 47011 Valladolid, Spain; arias@bio.uva.es; 3 BIOFORGE (Group for Advanced Materials and Nanobiotechnology), University of Valladolid, CIBER-BBN, LUCIA Building, Paseo de Belén, 47011 Valladolid, Spain; agirotti@eii.uva.es; 4 School of Optometry and Vision Science, University of Waterloo, Waterloo, ON N2L 361, Canada; denise.hileeto@uwaterloo.ca

**Keywords:** metronomic chemotherapy, cancer therapy, tumor microenvironment, tumor vascularization, bone-marrow-derived cells, cancer-associated fibroblasts, nanomedicine, nanocarriers

## Abstract

**Simple Summary:**

Metronomic chemotherapy with different mechanisms of action against cancer cells and their microenvironment represents an exceptional holistic cancer treatment. Each type of tumor has its own characteristics, including each individual tumor in each patient. Understanding the complexity of the dynamic interactions that take place between tumor and stromal cells and the microenvironment in tumor progression and metastases, as well as the response of the host and the tumor itself to anticancer therapy, will allow therapeutic actions with long-lasting effects to be implemented using metronomic regimens. This study aims to highlight the complexity of cellular interactions in the tumor microenvironment and summarize some of the preclinical and clinical results that explain the multimodality of metronomic therapy, which, together with its low toxicity, supports an inhibitory effect on the primary tumor and metastases. We also highlight the possible use of nano-therapeutic agents as good partners for metronomic chemotherapy.

**Abstract:**

The concept of cancer as a systemic disease, and the therapeutic implications of this, has gained special relevance. This concept encompasses the interactions between tumor and stromal cells and their microenvironment in the complex setting of primary tumors and metastases. These factors determine cellular co-evolution in time and space, contribute to tumor progression, and could counteract therapeutic effects. Additionally, cancer therapies can induce cellular and molecular responses in the tumor and host that allow them to escape therapy and promote tumor progression. In this study, we describe the vascular network, tumor-infiltrated immune cells, and cancer-associated fibroblasts as sources of heterogeneity and plasticity in the tumor microenvironment, and their influence on cancer progression. We also discuss tumor and host responses to the chemotherapy regimen, at the maximum tolerated dose, mainly targeting cancer cells, and a multimodal metronomic chemotherapy approach targeting both cancer cells and their microenvironment. In a combination therapy context, metronomic chemotherapy exhibits antimetastatic efficacy with low toxicity but is not exempt from resistance mechanisms. As such, a better understanding of the interactions between the components of the tumor microenvironment could improve the selection of drug combinations and schedules, as well as the use of nano-therapeutic agents against certain malignancies.

## 1. Introduction

The concept of cancer as a systemic disease encompasses the intercellular interactions between cancer cells and stromal cells and the non-cellular components of the microenvironment. These interactions are essential to support primary tumor growth and distant metastasis progression and may influence the therapeutic response [1,2].

Tumor hypoxia promotes acidosis and necrosis [3,4] and has been associated with abnormal angiogenesis, immunosuppression [5], tumor progression, and increased therapy resistance [6,7,8]. The heterogeneity in the vascular perfusion within the tumor arises as a result of the formation of abnormal tumor vessels, which leak and can be compressed by both cancer and stromal cells (e.g., desmoplastic tumors). The strategies to decrease tumor hypoxia through vascular normalization and normalization of cancer-associated fibroblast/extracellular matrix could improve the outcome of therapies [9,10,11,12,13]. In addition to the vascular network, other sources of heterogeneity and plasticity in the tumor microenvironment are the immune-infiltrated cells and the cancer-associated fibroblasts (CAFs) [1,14], which could have a dual role in tumor progression and metastasis [15].

Anticancer drug therapies induce host response effects that modify the tumor microenvironment, and these effects can enhance or reduce their therapeutic potential [16,17]. In this sense, the conventional chemotherapy regimen, at the maximun tolerated dose (MTD), which preferentially targets highly proliferative cancer cells, can induce cellular and molecular responses in the tumor and the host that can promote tumor regrowth, tumor cell dissemination, and metastasis [17]. In contrast, metronomic chemotherapy (MC), defined as the chronic administration of less toxic lower dosages of cytotoxic drugs with short or no drug-free breaks [18,19] and characterized by a multimodal effect on cancer cells and their microenvironment [20,21] shows limited induction of these host response effects [17]. As such, understanding these complex and multifaceted interactions could help improve the selection of drug combinations and schedules and reduce resistance to anticancer drugs. MC has become a new treatment option [22] that can be applied to chemotherapy, radiotherapy, and biotherapy [23]. In this regard, nanotherapy offers smartly targeted nanoparticles with modifiable properties to target components of the tumor microenvironment and could therefore be a good addition to MC [24].

In this review, we briefly define some cellular components of the tumor microenvironment and their reciprocal crosstalk to promote tumor progression, which could be affected by multimodal MC. We summarize the effects of host responses after MC regimen versus standard chemotherapy regimen and the general biological mechanisms of action of MC to explain its antitumor effects. Furthermore, we show that by using sophisticated nanocarriers to administrate the drug, the benefits of metronomic chemotherapy may be improved in terms of the selectiveness of drug delivery and therapy specificity.

## 2. Metronomic Chemotherapy: A Multimodal Therapy

The definition of MC as “a frequent, regular administration of drug doses designed to maintain low, but active, concentrations of chemotherapeutic drugs over prolonged periods of time, without causing serious toxicities” [25] highlights the importance of pharmacokinetic analysis, which provides information on optimal dosing and administration schedules for a single drug or in combination [25,26,27].

MC exhibits multiple potential mechanisms of action, which, together with its low toxicity, results in an inhibitory effect on both primary tumors and metastases [28,29]. The selection of the most appropriate drug or drug combinations, the administration schedule, and the tumor to be treated will affect the target of action, effectiveness, and resistance to therapy [30,31,32]. Thus, with an adequate selection of the above parameters, MC induces a selective antitumor immune response [33].

The concept of MC, and its therapeutic use, have been extensively reviewed [19,20,21,22,23,25] as regards the treatment of breast cancer [34,35], non-small-cell lung cancer (NSCLC) [36], and high-risk pediatric malignancies [37]. A systematic review on MC compiles the clinical experience in different cancers such as breast, castration-resistant prostate, ovarian, glial, renal, lung, gastrointestinal, hepatocellular, multiple myeloma, melanoma, and head and neck [38]. Moreover, MC, preferentially in a combination-therapy context, has demonstrated remarkable efficacy in post-surgical therapy models for advanced and early metastatic disease, especially for the treatment of several types of aggressive cancers [39]. In some cases, a differential response to therapy between metastasis and primary tumor was observed [40]. The efficacy and tolerability of MC when used as adjuvant in locoregionally advanced nasopharyngeal carcinoma patients [41,42], maintenance therapy in early-stage triple-negative breast cancer and high-risk rhabdomyosarcoma patients [43,44], and as treatment option in frail elderly patients with HER2-positive metastatic breast cancer [45] has been demonstrated in recent phase III trials. Two systematic reviews of the literature of metronomic clinical trials, covering the periods 2000/2012 and 2012/2019, showed that most of the metronomic studies refer to breast, lung, malignant glioma, and prostate cancer, with the most widely used being the cytotoxic drugs cyclophosphamide, capecitabine, vinorelbine, methotrexate, and temozolamide, mainly in combination with other therapies and mostly using empirical doses and schedules. These reviews conclude that although MC is active and improves quality of life, further such trials are required to confirm its widespread clinical utility [46,47].

MC is not exempt from resistance processes. However, they develop more slowly compared to the MTD regimen, which could be due to the multimodal character of the former [20,48]. In this regard, it has been demonstrated that MC modulates the balance of drug-resistant and drug-sensitive clones co-existing within a tumor better than an MTD regimen [49]. Cancer cells and host cells develop tumor support mechanisms to escape therapy. Therefore, the drivers and targets for overcoming resistance to MC and other antiangiogenic therapies are found in both cancer cells and host cells [48,50,51].

## 3. Cellular Components of the Tumor Microenvironment and Their Modulation by Metronomic Therapy

### 3.1. Tumor Vasculature as a Support for Cancer Cells and Tumor-Initiating Cells or Cancer Stem Cells (CSCs)

The normal vascular tree is a heterogeneous and highly organized network comprising both supply and drainage vessels. The lining endothelial cells, especially capillary endothelial cells, regulate the crosstalk between the needs of the tissue and the overall systemic circulation, exhibiting differential gene expression in different tissues and organs, and even within the same tissue [52,53,54].

During tumor progression, a vascular network is formed and remodeled as a result of different processes that can coexist in tumors, such as de novo arteriogenesis, venogenesis [55,56], sprouting angiogenesis, initiation from capillaries, and vasculogenesis, with bone-marrow-derived endothelial progenitor cells (EPCs) being involved [57]. In addition, vessel co-option and vasculogenic mimicry are non-neoangiogenesis mechanisms of vessel formation in tumors. Vessel co-option is a mechanism by which tumor cells grow around pre-existing blood vessels, mainly observed in primary and metastatic lung, liver, and brain tumors, as well as in lymph nodes, and it requires motility and invasion of cancer cells [58,59]. In the vasculogenic mimicry mechanism, CSCs participate in the formation of vascular-like structures. This mechanism is associated with higher tumor invasiveness and recurrence [60,61]. Both mechanisms mediate resistance to anti-angiogenic therapy [62,63].

The microenvironment of a growing tumor is characterized by high heterogeneity in terms of hypoxia and acidity levels, and by the continuous production of pro-angiogenic factors by tumor and stromal cells [64,65]. The tumor vascular network is formed and remodeled with no spatio-temporal control, thus exhibiting significant heterogeneity in terms of structure, organization, and function [56,66].

The different characteristics of tumor endothelial cells (TECs) and normal ECs have recently been reviewed [67]. TECs establish communication with tumor cells and other stromal cells via the release of angiocrine factors that control tumor progression [68]. Angiocrine signaling from endothelial cells plays a role in maintaining disseminated tumor cells, which reside in perivascular niches of the metastatic place, in a dormant state [69,70]. In addition, pericytes perform regulatory functions in vessel stabilization, permeability, and blood flow; play a role in the angiogenesis process; and interact with endothelial cells, tumor cells, cancer-associated fibroblasts (CAFs), and immune cells to promote tumor growth and progression [71].

CSCs may contribute to blood vessel formation by transdifferentiation into endothelial cells or pericytes [60]. Hypoxic peri-arteriolar glioma stem cell niches exist in human glioblastoma samples, with the SDF-1α/CXCR4 signaling axis and osteopontin/CD44 interactions being involved in the homing of glioma stem cells in their niches and their maintenance [72,73]. Arterioles are transport vessels, not exchange vessels like capillaries [73]. In an in vitro sphere-forming assay with C6-rat-glioma cell line, which contains brain tumor stem-like cells, it was confirmed that factors secreted by human umbilical vein endothelial (HUVEC) cells significantly increased the number of primary tumor spheres formed [74]. Similarly, in an in vivo study, C6-rat-glioma tumor xenografts with low and high CSCs were compared. A higher microvessel density and blood perfusion was found in the latter, along with an increase in bone-marrow-derived EPCs in the bloodstream and their incorporation into the vessels [75].

#### Metronomic Chemotherapy to Target Endothelial, Cancer, and Cancer Stem Cells

A systematic review of preclinical studies has confirmed the significant relationship between reduced tumor vascularization and inhibition of tumor development, thus highlighting the importance of vascular efficiency [76]. However, a microvascular density analysis cannot distinguish between angiogenic and non-angiogenic tumors, therefore the histopathological growth pattern of tumors must be taken into account [77] or the term “microvessel density” redefined [78,79]. Two tumor phenotypes have been distinguised in murine tumor models and tumor specimens from patients based on the vascular/stromal architecture ratio, which are known as “tumor vessel” and “stromal vessel” and are sensitive or refractory to the VEGFR-2-blocking antibody DC101, respectively [80].

The anti-angiogenic potential of MC has been recently reviewed [81] and, given that tumor cells and CSCs depend on the tumor vasculature, they will be affected. Prolonged and continuous exposure in vitro to low doses of chemotherapy drugs has shown that much lower concentrations of paclitaxel or 4-hydroperoxycyclophosphamide promote the inhibition of proliferation and the induction of apoptosis in active endothelial cells more than in tumor cells [82]. Following the same in vitro assay, a protracted low dose of vinorelbine showed a direct anti-proliferative effect on NSCLC cells [83].

Unlike the administration of high doses of drugs, MC treatment showed a delayed but sustained antitumor effect and a reduced resistance to therapy, which was explained by its antiangiogenic effects and the activation of antitumor immunity [19,84]. In the combination of MC and a conventional antiangiogenic drug, the antitumor efficacy was mutually enhanced [19,85]. There is evidence from mouse tumor models and clinical trials that MC decreases the levels of proangiogenic factors and increases the levels of endogenous angiogenesis inhibitors, such as trombospondin-1 (TSP-1), which may contribute to the angiogenic dormancy of the primary tumor [86].

In a PET-MRI study in recurrent glioblastoma patients treated with [^18^F]FMISO, a PET tracer incorporated by viable hypoxic cells and not affected by perfusion, before and after treatment with bevacizumab, an intrinsic resistance to bevacizumab was observed in certain hypoxic tumor regions, as shown by an increased cerebral blood volume/cerebral blood flow ratio and larger vessels compared to non-hypoxic regions. Those regions whose initial hypoxia was reversed by the treatment showed shorter mean transit times, thus demonstrating vascular normalization and improved oxygenation [87].

Vascular normalization combined with chemotherapy or radiotherapy has emerged as a new antitumor therapy, although numerous factors must be taken into account during optimization [10]. Mathematical modeling has been used to define the normalization window for increased perfusion after bevacizumab administration, a period in which the release of a cytotoxic drug could be increased. In this way, the optimal schedule for sequential administration of bevacizumab and pemetrexed-cisplatin in human NSCLC xenograft model has been defined [88] and scaled for use in humans [89]. A mathematical model has also been used to assess the value of metronomic therapy in a vascular normalization strategy [90]. Thus, using a patient-derived xenograft of a pancreatic cancer model, treatment with metronomic gemcitabine caused a cytostatic effect, that is, viable but non-proliferative tumor cells, an improvement in tumor perfusion, a reduction in hypoxia and necrosis, and a decrease in tumor metabolism compared to the control [91].

In addition to the antiangiogenic effects of MC, in a sphere-forming assay using C6-rat glioma xenografts, Folkins et al. demonstrated that metronomic cyclophosphamide alone, or MTD cyclophosphamide + DC101, reduces the number of tumor spheres by targeting CSCs [74]. Two metronomic schedules of gemcitabine treatment in orthotopic human pancreatic tumor models reduced the percentage of pancreatic CSCs subpopulations [92].

### 3.2. Tumour Microenvironment May Reprogram Non-Immunological Bone Marrow-Derived Cells to Support Tumor Growth and Metastases

Tumor hypoxia induces tumor vascularization by promoting the production of angiogenic factors by tumor and stromal cells [93] and acts as a trigger in bone-marrow-derived cell (BMDC) recruitment, a complex process involving mobilization, peripheral blood circulation, transendothelial migration, and tumor homing [94,95,96]. The BMDCs recruited by the tumor include CD133^+^/CD34^+^/VEGFR-2^+^ bone-marrow-derived endothelial progenitor cells (EPCs), which undergo differentiation and incorporation into newly formed blood vessels, where they stimulate tumor vascularization as a result of paracrine signaling [95,97,98]. On the other hand, several subsets of hematopoietic progenitor cells (HPCs) recruited into the tumor bed act as peri-vascular modulators by producing growth factors, cytokines, and matrix metalloproteinase-9 (MMP9), a critical molecule for vascular remodeling and neovascularization [97]. The homing of EPCs to the tumor bed is regulated by several chemokines and their receptors, especially VEGF/VEGFR-2 and SDF-1α/CXCR4 [95]. Given the complexity of the research on the origin, characterization, and contribution of EPCs to neovascularization, it has yielded widely differing results [98,99].

The contribution of EPCs to tumor vasculogenesis was demonstrated using angiogenesis-defective Id mutant (Id1^+/−^Id3^−/−^) and wild-type mice inoculated with human lymphoma and Lewis lung carcinoma (LLC) cells. The tumors obtained from wild-type mice were well vascularized, unlike those obtained from mutant mice, which did not show blood vessel infiltration and presented necrosis and very slow growth [100]. When Id mutant mice were lethally irradiated, transplanted with wild-type bone marrow, and then inoculated with tumor cells, tumor growth was restored. VEGFR-2^+^-circulating endothelial progenitor cells (CEPs) were incorporated into the neo-vessels and surrounded by VEGFR-1^+^-HPCs, which confer stability [101]. A high expression of Id1 and Id3 has been found in many types of cancer, both in the vasculature and in tumor cells [102].

Higher levels of VEGFR-2^+^-CEPs were found in the blood of patients with recurrent or metastatic pediatric solid malignancies [103]. Using reconstituted bone-marrow mice injected with LLC cells, a sequential implication of VEGFR-1^+^-HPCs and VEGFR-2^+^-CEPs in the formation of micrometastasis was shown. Anti-VEGFR-1-antibody treatment prevented the formation of lung pre-metastatic VEGFR-1^+^-HPCs clusters, consequent tumor cell recruitment, and metastasis formation, and anti-VEGFR-2-antibody treatment prevented large, well-vascularized metastases [104,105]. An elevation of VEGFR-1^+^-HPC levels was observed in blood samples from patients with breast cancer, with an average time of 6 months before relapse, and a subsequent elevation of VEGFR-2^+^-CEPs one month before relapse, thus suggesting that they represent the early stages of development of metastasis and treatment opportunities [106]. Moreover, there is some clinical evidence for CEPs in patients with solid and hematological cancers [107,108].

#### Metronomic Chemotherapy Has a Systemic Antiangiogenic Effect by Reducing Mobilization and Viability of Bone-Marrow-Derived CEPs

The levels of VEGFR-2^+^-CEPs and mature circulating endothelial cells (CECs), the latter possibly derived from tumor endothelium by vascular turnover [109], found in peripheral blood before and after tumor treatment could help to explain vascularization and tumor progression and the biological activity of a therapeutic agent [110,111]. In this regard, a systematic study of the clinical diagnostic and prognostic value of CEP levels concluded that, although higher CEP levels were found in the blood of cancer patients than in healthy subjects, a deeper understanding of these cells and the tumor vascularization process is required before CEPs can be used as tumor biomarkers [112].

Children with metastatic disease or relapses presented higher CEP levels compared to healthy subjects [103]. In addition, a reduction in blood CEP levels was detected in children with acute lymphoid leukemia (ALL) 6 months after the start of standard maintenance chemotherapy, with characteristics of metronomic therapy, and was maintained over time. Moreover, a significant increase in circulating levels of thrombospondin-1 was observed after 18 months of treatment [113]. In patients with advanced breast cancer, after two months of metronomic chemotherapy (cyclophosphamide/methotrexate +/- thalidomide), an elevation of apoptotic blood CECs was observed and found to be correlated with a better clinical outcome after follow-up of the patients for two years [114]. In a trial of lenalidomide and metronomic melphalan in elderly patients with chronic myeloid leukemia, it was observed that baseline CEC levels in responding patients were higher than in non-responders, and that peaks of CECs occurred more frequently than in patients with progressive disease [115]. A trial of metronomic vinorelbine and sorafenib, as palliative treatment in patients with advanced NSCLC, showed a better response at lower doses. Moreover, dynamic changes in CECs occurred during treatment, and their overall increase was a predictor of better survival [116].

In human lymphoma xenograft models, CEP levels were observed to increase in parallel with tumor growth. A peak in blood CEP levels occurred a few days after MTD cyclophosphamide treatment, thus resulting in tumor resistance. In contrast, metronomic cyclophosphamide produced a significant and sustained reduction in the number and viability of CEPs and a delay in tumor growth [117]. The acute host response to MTD treatment was not generally mediated by the different chemotherapeutic drugs tested [118].

The administration of Oxi-4503, a vascular disrupting agent (VDA), promoted an acute elevation of viable CEPs in blood. A high concentration of GFP^+^-CEPs was found to be localized at the tumor site, with some being incorporated into the peripheral tumor vasculature. Administration of DC101 before VDA avoided these effects [119,120], similar to MC cyclophosphamide treatment, thus resulting in a sustained delay of primary tumor growth associated with lower perfusion and an increased apoptosis of tumor cells [121].

The optimal biological dose (OBD) for a therapeutic drug is defined as the lowest dose that causes the maximum antitumor effect and safety. A correlation between OBD and the maximum decrease in viable blood CEPs caused by antiangiogenic drugs and several chemotherapy drugs administered in a metronomic regimen has been demonstrated [122,123].

Two combination treatments, namely endostar with metronomic vinorelbine or endostar with vinorelbine at the MTD, were assayed in an LLC mouse xenograft model. These treatments affected CEP mobilization, tumor vessel number, and tumor expression of VEGF and hypoxia inducible factor-1 (HIF-1α) differently. Thus, whereas the former promoted a significant decrease, the latter showed an increase in comparison with the control group [124]. There is some clinical evidence for the efficacy and safety of metronomic vinorelbine in advanced breast cancer and NSCLC [125,126]. Metronomic topotecan with pazopanib showed antitumor activity in murine models of aggressive pediatric solid tumors and a significant reduction in viable blood CEPs and CECs and tumor microvessel density [127]. Treatment with MTD capecitabine, as monotherapy or combined with metronomic cyclophosphamide, increased HIF-1α in primary orthotopic human metastatic colon adenocarcinoma and in its liver metastasis. Substitution with metronomic capecitabine decreased HIF-1α levels and was correlated with a decrease in intra-metastatic hypoxia and nodule size [128].

### 3.3. Tumour Microenvironment May Reprogram Immunological Bone-Marrow-Derived Cells to Support Tumor Growth and Metastases

Myeloid-derived suppressor cells (MDSCs) CD11b^+^GR1^+^ in mice are immature bone marrow myeloid cells characterized by immunosuppressive activity [129] and heterogeneity [130]. Factors produced by cancer and stromal cells, such as granulocyte-colony-stimulating factor (G-CSF) and granulocyte-macrophage-stimulating factor (GM-CSF), promote the expansion of immature myeloid cells (IMCs) in the bone marrow and their migration to, and accumulation in, peripheral lymphoid organs and tumor sites [131]. IMCs Cd11b^+^Gr-1^int^Ly6C^hi^ accumulate in the marginal region of the spleen and expand during tumor development [132]. A plethora of factors at tumor sites trigger different signaling pathways in MDSCs that lead to the development of immature monocytic myeloid-derived suppressor cells (M-MDSCs) Cd11b^+^Gr-1^hi^Ly6C^lo^ and polymorphonuclear myeloid-derived suppressor cells (PMN-MDSCs) Cd11b^+^Gr-1^lo^Ly6C^hi^. The induction of immunosuppressive pathways in MDSCs inhibits tumor T-lymphocyte responses via several mechanisms and promotes regulatory T cells (Treg) [133]. Apart from MDSCs, tumors drive the recruitment and infiltration of other myeloid cells, such as macrophages, neutrophils, and dendritic cells [131]. Immunosuppressive pathways can operate in these other myeloid cell types, thus resulting in macrophage and neutrophil polarization to a pro-tumor phenotype and the blocking of dendritic activation [133].

Tumor-associated MDSCs and macrophages (TAMs) are key components of CSC niches. Both release cytokines, inflammatory molecules, and growth factors that support CSCs [134]. A cross-talk is established between MDSCs/TAMs and CSCs in the TME. Thus, MDSCs were found in the TME from glioblastoma patient samples, and a significant fraction of them were found close to CSCs [135]. In studies with glioblastoma xenograft tumors and co-culture strategies, it was demonstrated that macrophage migration inhibitory factor (MIF), which is secreted at high levels by CSCs, activated the immunosuppressive phenotype of MDSCs in a CXCR2-dependent manner [135].

Tumor-associated MDSCs promote tumor progression by inducing tumor angiogenesis via the release of growth factors, cytokines, and metalloproteinases (e.g., VEGF, Bv8, MMP9) in response to tumor hypoxia and by STAT3 activation in MDSCs. Similarly, tumor-associated MDSCs can be transdifferentiated into endothelial-like cells. The VEGF produced stimulates the recruitment of MDSCs by the tumor, thus creating an immunosuppressive and antiangiogenic positive feed-forward loop [136]. In addition, recruitment of CD11b^+^Gr1^+^ cells has been associated with refractoriness to anti-VEGF therapy [137,138].

MDSCs can promote tumor invasion and are essential for the formation of a pre-metastatic niche [139,140,141]. In this regard, MDSC levels were found to be significantly higher in the blood and tumors of patients with different types of cancers than in healthy individuals, and were correlated with the clinical cancer stage and metastatic tumor burden [142,143,144,145]. Recent studies, in the context of clinical trials, have suggested a correlation between circulating and intratumoral MDSC levels and tumor stage, progression, and resistance to therapy [146]. However, due to the phenotypic complexity of these cells, which is governed by the microenvironment, a greater number of clinical trials and standardized studies remains necessary [147,148,149,150].

A small subset of circulating CD11b^+^-myeloid cells expressing the Tie-2 angiopoietin receptor, known as Tie-2 expressing monocytes (TEM), which are a subset of tumor-associated macrophages (TAM), was identified, along with their infiltration into orthotopic tumors and perivascular location [151]. TEMs have pro-angiogenic potential by secreting angiogenic factors and high levels of MMP9 [151,152]. In cancer patients, TEMs were found in the blood and in different types of solid tumors, usually close to the blood vessels [153]. Tumor-derived angiopoietin-2 (Ang-2) induces chemotactic activity in TEMs [152,153], which have been associated with resistance to therapies that block VEGF and VEGFR-2 and with the development of an invasive phenotype [154,155].

#### Metronomic Chemotherapy as Immune Modulator by Targeting Bone-Marrow-Derived Myeloid Cells

Treatment of mice bearing metastatic pancreatic adenocarcinoma with MTD gemcitabine significantly increased the mobilization and tumor infiltration of MDSCs expressing high levels of the pro-angiogenic chemokine Bv8, thus resulting in highly perfused tumors and rapid tumor regrowth [156]. When MTD treatment was combined with metronomic gemcitabine, or with anti-Bv8-antibodies, the number of MDSCs in the tumor was significantly reduced, which resulted in a significant reduction in angiogenesis, tumor regrowth, and metastasis [156]. In addition, in an aggressive mice model of pancreatic adenocarcinoma, treatment with MTD gemcitabine after tumor resection resulted in a reduction of local recurrences, but not distal metastases, mainly due to an increased infiltration of natural killer cells (NK) at the resection margin [157]. The neoadjuvant and adjuvant modality of chemotherapy could be modulated by the tumor microenvironment and host conditions [141].

In brain tumor xenograft models, metronomic cyclophosphamide (Q6day cycle) activated anti-tumor immunity (blocked by axitinib and DC101), which is associated with tumor regression, and decreased CD11b^+^GR1^+^ reservoirs in bone marrow and spleen [158,159]. In a mouse glioma model, metronomic 5-FU produced a selective reduction in blood MDSC levels, which is associated with an increase in CD8^+^-T cells and a reduction in Treg, which attenuated tumor immunosuppression and prolonged survival, whereas a higher dose did not [135]. A similar result was obtained in breast carcinoma patient-derived xenograft mouse models treated with capecitabine using MTD or metronomic regimens [160]. In patients with recurrent glioblastoma, treatment with metronomic capecitabine prior to surgery produced a decrease in circulating MDSCs and an increase in tumor infiltration of CD8^+^-T cells and NK immune cell populations [161,162].

In an anti-VEGF-refractory LLC ectopic model, post-surgical treatment with anti-Ang-2 antibodies inhibited the growth of lung metastases and reduced the vessel area in metastatic nodules. The combination of metronomic gemcitabine/anti-Ang-2/anti-VEGF therapy showed a very significant reduction in metastatic burden [163]. This therapeutic effect was explained by the blocking of MDSC recruitment to metastasis, together with inhibition of the adherence of pro-metastatic macrophages to endothelial cells and their infiltration at the metastasis site [163].

Tregs is another type of immunosuppressive cell in the TME [164] whose plasticity and function have been reviewed recently [165]. Several studies have indicated a potential role for Treg in tumor angiogenesis [166,167]. As such, antiangiogenic drugs may affect the number and function of Treg [168]. In a rat glioma model, metronomic temozolomide selectively and significantly decreased the Treg/CD4^+^ ratio in the spleen [169]. Clinically, elderly breast cancer patients treated for 6 months with letrozol or letrozol + metronomic cyclophospamide showed a significant reduction in tumor Tregs associated with a good response to treatment [170]. Similarly, an increase in tumor-infiltrating lymphocytes was observed in breast cancer patients treated pre-operatively for 3 weeks with letrozole or letrozol + metronomic vinorelbine [171]. Treatment of patients with metastatic solid tumors with metronomic cyclophosphamide for one month induced a strong decrease in blood levels of Tregs and restored immune functions [172]. In a phase I/II trial of metronomic cyclophosphamide in patients with metastatic colorectal cancer, a depletion of Tregs in the blood was observed in parallel with an increase in CD8^+^-T cells and a significantly longer progression-free survival [173].

Metronomic chemotherapy exerts immune-modulatory effects, unlike the standard chemotherapy regimen; for this reason, it was proposed to combine it with immunotherapies, such as anti-CTLA-4 and anti-PD-1, the immune checkpoint inhibitors [174], to enhance its effects in overcoming the cancer-induced immunosuppressive tumor microenvironment [31,34,175,176]. In this sense, metronomic paclitaxel combined with programmed cell death 1 (PD-1) mAb in a syngeneic breast cancer mouse model improved the anti-tumor efficacy of the immune checkpoint inhibitor as monotherapy, with a significant benefit in survival and reduced toxicity. Analysis of the immune microenvironment of the tumor showed a reduction in the levels of Treg and MDSCs and an increase in the levels of CD4^+^ and CD8^+^ T cells [177]. In a murine glioma model, standard treatment with temozolomide promoted the exhaustion of CD4^+^ and CD8^+^ T-cell and an increase in Treg and MDSC levels. These effects were not observed with metronomic temozolomide. The combination of the metronomic temozolomide regimen with PD-1 mAb preserved but did not increase the beneficial effects of PD-1 inhibition, effects that were abolished by the combination with the standard dose of temozolomide [178]. In a preclinical study using an EMT6/P breast cancer mouse model, blocking cytotoxic T-lymphocyte antigen 4 (CTLA-4) inhibited tumor growth. This inhibitory effect was increased when the anti-CTLA-4 antibody was combined with a low-dose cyclophosphamide regimen, but not with a bolus (high-dose) injection of cyclophosphamide plus a low-dose cyclophosphamide regimen [179]. In another preclinical study, the metronomic cyclophosphamide regimen (Q6day cycle) inhibited tumor growth and significantly increased survival in mice with aggressive EMT6-CDPP breast cancer tumors. Analysis of the treated tumors showed an increase in CD4^+^ and CD8^+^ T lymphocytes and B lymphocytes. Although this cyclophosphamide therapy regimen was shown to increase PD-L1 expression on the surface of EMT6-CDPP tumor cells, its combination with anti PD-L1 antibody did not show greater efficacy compared to anti-PD-L1 antibody single-agent [180]. In a phase II clinical trial (TONIC), short-term sequential administration of metronomic doxorubicin, or cisplatin but not cyclophosphamide, as induction treatment, followed by nivolumab (anti-PD-1) increased objective response rates (ORRs) in patients with triple-negative breast cancer [181]. In this sense, a phase II trial that compared the efficacy of a combined biomodulatory therapy (pioglitazone + clarithromycin + low-dose metronomic treosulfan) with nivolumab (anti-PD-1) as a single-agent in patients with locally advanced, unresectable, or metastatic NSCLC showed that biomodulatory therapy was well tolerated and showed stabilization of the disease. Although progression-free survival (PFS) in the biomodulatory treatment arm was significantly lower than for the nivolumab treatment, both treatments showed similar overall survival (OS). Based on these results, it was suggested that biomodulatory therapy could be used as an induction treatment to improve the efficacy of immune checkpoint inhibitors [182]. All these data highlight the difficulty and complexity of improving the efficacy of immune checkpoint therapy with metronomic chemotherapy as an immune-modulator. With this goal in mind, essential factors such as tumor type, drug type, dose, timing, treatment schedule, and method of drug administration must be considered and optimized.

### 3.4. Heterogeneity and Plasticity in Cancer-Associated Fibroblasts (CAF), a Subset of the Tumor Microenvironment

CAFs, which are a major cell population in the tumoral stroma, are phenotypically and functionally heterogeneous and are not completely characterized [183,184]. Their heterogeneity may depend on the multiplicity of origins or their different spatial distribution inside the tumor [185,186,187]. In addition, resident endothelial cells may undergo an endothelial-to-mesenchymal transition to CAFs with migratory potential, thus playing a role in the metastatic process [188,189].

CAFs have an active and dynamic secretome that is involved in angiogenesis, tumor immunity, migration and invasion, resistance to therapy, and tumor recurrence [190,191,192]. CAFs together with tumor cells participate in the deposition and remodeling of the extracellular matrix (ECM). Indeed, CAFs secrete ECM-degrading proteases (e.g., matrix metalloproteinases) that release matrikines and growth factors from the ECM reservoir, promoting tumor growth and metastasis [193]. On the other hand, the degradation of the ECM by matrix metalloproteinases (MMPs) favoring lymphocyte tumor infiltration and immunomodulation of the tumor microenvironment shows how MMPs play both pro-tumorigenic and anti-tumorigenic roles [194]. Several trials have focused on therapies based on the MMPs’ inhibition, and even if unsatisfactory results have been described so far, they have contributed to emphasizing the complexity of the metalloproteases family [195]. CAFs may mediate resistance to androgen-deprivation therapy, which is effective in the treatment of prostate cancer. Prostate cancer cells and CAFs express the androgen receptor (AR), and when the AR signaling is therapeutically inhibited in CAFs, it increases the expression and release of both CCL2 and CXCL8 cytokines, which exert pacracrine signaling in cancer cells of the prostate, promoting cell migration and invasion [196]. Viewing tumors as “wounds that never heal” [197] allows us to consider CAFs as permanently activated fibroblasts associated with tumors [198]. However, there are different CAF subsets, even within the same tumor, with different phenotypes and levels of activation.

In one experiment, MCF-7-ras breast cancer cells combined with human CAFs (or normal fibroblasts as control) were grafted into SCID mice, and the SDF-1 produced by CAFs was found to increase tumor mobilization and EPC recruitment [199].

In a multi-step squamous skin carcinogenesis model, fibroblasts isolated in the early hyperplastic stage exhibited a pro-inflammatory gene signature (e.g., chemokines CXCL1, SDF-1, IL6 cytokine, osteopontin) that persists during all stages of cancer progression. In this step, IL1-β expressed by resident activated immune cells promotes CAF induction via NF-κB signalling, thereby resulting in a proangiogenic and tumor-promoting inflammatory response as a result of the recruitment of macrophages into the tumor [200].

Costa et al. characterized four CAF subsets in samples from breast cancer patients at the time of surgery before any treatment. The subsets CAF-S1 (CD29^Med^FAP^Hi^FSP1^Med^αSMA^Hi^PDGFRβ^Med−Hi^CAV1^Low^), the only one positive for fibroblast activation protein-α (FAP), and CAF-S4 (CD29^Hi^FAP^Neg^FSP1^Low−Med^αSMA^Hi^PDGFRβ^Low−Med^CAV1^Low^) preferentially accumulate in the most aggressive triple-negative breast cancer subtype. These subsets promote an immunosuppressive microenvironment by attracting CD4^+^CD25^+^-T lymphocytes, increasing their survival, and promoting their differentiation into immunosuppressive CD25^+^FOXP3^+^ Treg cells [201].

FAP^+^-CAFs were isolated by digestion of murine hepatoma tumor tissues and found to be an important source of the chemokine CCL2, which mediates tumor inflammation and immunosuppression. STAT3-CCL2 signaling, which is activated by FAP, promoted tumor growth by enhancing the recruitment of MDSCs (CD11b^+^Gr1^+^) and macrophages, both of which express the CCR2 receptor, thus preventing antitumor IFNγ-T-cell immunity. A significant positive correlation was found between the expression of FAP, pSTAT3, and CCL2 in the tumor stroma in patients with intrahepatic cholangiosarcoma, a highly aggressive primary desmoplastic tumor associated with poor overall survival and a high probability of recurrence [202].

A new CD10^+^GPR77^+^-CAF subset associated with poor survival was identified in patients with breast and lung cancer. This subset is abundant in chemoresistant tumors and provides a survival niche for CSCs as a result of continuous paracrine secretion of IL6/IL8 [203,204]. A pro-tumorigenic integrin-α11^+^/PDGFRβ^+^-CAF subtype has recently been identified in human breast cancer tissues associated with metastasis and poor clinical outcome. Furthermore, integrin-α11 plays a role in the regulation of PDGFRβ signaling, leading to JNK activation and a subsequent increase in tenascin c [205].

#### Metronomic Chemotherapy Prevents the Pro-Stemness Function of CAFs

A better understanding of the functional, spatial, and temporal heterogeneity of CAFs, and the presence of subsets with opposite effects on cancer progression, will allow us to develop more specific, effective, and safe therapies [206], thereby avoiding the adverse effects observed after complete depletion of CAFs [207,208,209].

Due to the presence of pro-stemness CAFs, it is essential to consider the dose and timing (neoadjuvant or adjuvant setting) of administration of CAF-targeted therapy [210]. Neoadjuvant MTD chemotherapy (doxorubicin, paclitaxel, or cyclophosphamide) induced the expression of ELR^+^-chemokines in CAFs from human breast cancer tissues and in breast and pancreatic ductal carcinoma xenograft models. Secreted ELR^+^-chemokines signal using the CXCR-2 receptor, in a paracrine manner, thus promoting tumor progression by stimulating angiogenesis, macrophage tumor infiltration, and phenotypic conversion and expansion of cancer cells to CSCs (CD44^+^CD24l^ow/−^) [211]. A significant increase in the percentage of CSCs was observed in biopsies from breast cancer patients after 12 weeks of preoperative treatment with docetaxel or doxorubicin and cyclophosphamide at standard doses [212]. Significantly, MC with the same chemotherapeutic agents attenuates ELR^+^-chemokine CAF expression and prevents therapy-induced expansion of CSCs. As such, it could be suitable for the treatment of desmoplastic tumors (e.g., breast, pancreatic cancer) with high levels of CAFs [28,211].

Pancreatic ductal adenocarcinoma has an intrinsic resistance to gemcitabine [213]. Orthotopic injection of pancreatic cancer cells combined with pancreatic stellate cells treated with MTD-gemcitabine into mice promoted a very rapid development of metastases. However, co-injection with pancreatic stellate cells treated with metronomic gemcitabine led to tumor regression and longer survival [211]. In human epidermoid carcinoma and NSCLC tumor-bearing mice, treatment with MTD paclitaxel monotherapy, in sensitive and drug-resistant mice, promoted extensive tumor infiltration of CAFs, which was increased in resistant tumors. Treatment of taxol-resistant human tumor xenografts with metronomic 5-FU in combination with MTD paclitaxel reverses tumor drug resistance by down-regulating P-glycoprotein, a multidrug efflux transporter, and decreasing density of CAFs and collagen in the tumor microenvironment [214].

**Figure 1:**
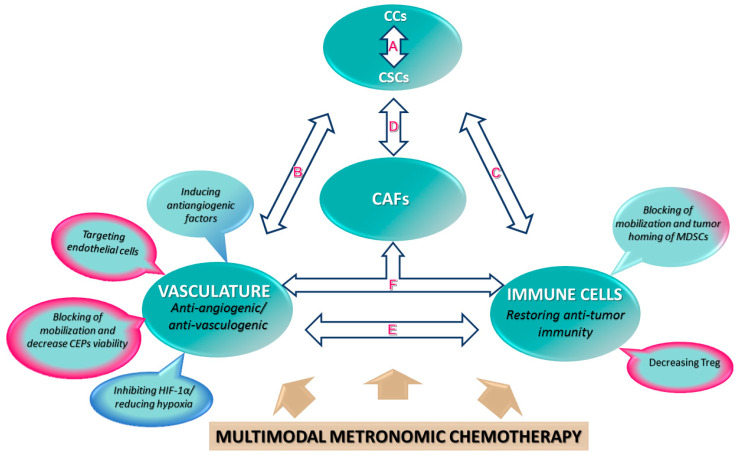
General biological mechanisms of action of MC to explain their antitumor effects. Links that create a permissive tumor microenvironment and could be affected by metronomic chemotherapy (two-way arrows). The CSC population is characterized by heterogeneity, plasticity, and therapy-resistance [215,216]. The reciprocal interactions between CSCs, cancer cells, endothelial cells, immune cells, CAFs, and other extracellular elements of the tumor microenvironment regulate CSCs in the primary tumor and metastases [217,218]. (**A**–**D**): (**A**) A feedback-signaling mechanism occurs between cancer cells (CCs) and CSCs. CSCs can induce the conversion of non-stem cells into metastatic-CSCs with a migratory phenotype [219]. Hypoxia/HIF-1α promotes epithelial-to-mesenchymal transition in cancer cells and maintains stemness [220]. (**B**) CSCs are drivers of vascularization, and the perivascular area constitutes a supportive niche. (**C**) CSCs maintain populations of immunosuppressive cells (e.g., MDSCs, Treg), and tumor-associated MDSCs, in turn, are key components of CSC niches and are essential for the formation of the pre-metastatic niche. (**D**) CSCs induce the transformation of normal stromal fibroblasts into CAFs, which maintains the proliferation and self-renewal of CSCs. Bidirectional link between vasculature and immune cells: (**E**) Tumor-recruited MDSCs are pro-angiogenic and can be transdifferentiated into endothelial-like cells. Tumor MDSCs are immunosuppressive by inhibiting tumor T-lymphocytes and NK cell activation and by inducing immunosuppressive Treg which, in turn, can promote tumor angiogenesis. Cross-talk between CAFs and the vasculature and immune system: (**F**) CAFs contribute to tumor angiogenesis and promote tumor CEP mobilization and recruitment. In turn, tumor endothelial cells can undergo an endothelial-to-mesenchymal transition to CAFs. In addition, CAFs mediate tumor immunosuppression by promoting MDSC recruitment and attracting T-lymphocytes and their differentiation into immunosuppressive Treg. This figure is based on previous studies [20,28]. See Section 3 for more detailed information. Reprinted with permission from the original with modifications Ref. [29], Copyright 2021, Elsevier.

In Figure 1 and Table 1, some of the most relevant results described in Section 3 are summarized.

## 4. Simulated Metronomic Therapies: Nanocarriers for Cancer Therapy

Advanced therapies for the clinical treatment of cancer are currently focusing on personalized medicine approaches by refining drug-delivery procedures and improving the specificity of therapy [221]. In this perspective, we show that most sophisticated pharmacological vehicles play an important role in increasing the bioavailability of drugs and their selectivity by delivering them directly to the tumor microenvironment and allowing controlled release once in situ [222,223]. The aim of metronomic therapy to administer a reduced but prolonged dose coincides with one of the main benefits of drug carriers [224]. The development of vehicles that “package” therapeutic agents allows the delivery of a lower dosage in a “nanometronomic” manner [225], while overcoming the obstacles presented by free drugs as regards alleviating systemic toxicity and side effects, increasing tumor penetration, and sustained drug release [226,227,228]. Indeed, by choosing the most suitable pharmacological vehicle for the therapeutic agent, the latter can be protected, its solubility increased, and its release modulated by prolonging the time of circulation and reducing the dosage [229].

Several drug-delivery systems, such as drug depots [230,231,232], hydrogels [233,234,235,236], microspheres [237,238], or nanocarriers (NCs) [239,240], have been developed and investigated. For intra-tumoral drug delivery and for bypassing physiological barriers, local drug-loaded depots and hydrogels have allowed the long-term delivery of treatment at the site of action [234,241]. Moreover, these devices reduce the frequency of administration and, therefore, patient discomfort, as only a single in situ application is required. Unfortunately, many tumors cannot be treated in situ with these macroscopic devices due to the possible inability to administer them in the target organ, or the danger inherent in doing so, and when the simultaneous treatment of different organs is necessary, such as in the case of metastatic tumors [242]. In contrast, nanocarriers can overcome these limitations as the administration of drug-loaded NCs can be systemic or localized, and they can cross physiological barriers, thus reaching widely disseminated cancer cells [243]. The benefits of NC-based pharmaceutical formulations are even more evident in cancers with poor prognosis, immunogenicity, and high resistance to current therapies, such as pancreatic [244,245], triple-negative breast [246], or ovarian cancer [247,248]. NC treatments have recently been approved, as they have been shown to provide survival benefit to patients with advanced aggressive cancers, or are currently undergoing active clinical trials [249,250,251]. Some of these therapies include combined treatments administrated in a metronomic manner [252]. Thus, the treatment of pancreatic ductal adenocarcinoma using a combination of gemcitabine-based therapy with irinotecan-, fluorouracil-, and folinic-acid-loaded NCs has shown anti-tumor efficacy and improved patient survival [249]. The effectiveness of the formulation known as CRLX101, which comprises self-assembling NCs formed from a cyclodextrin-containing polymer conjugated to camptothecin, against ovarian cancer is currently being studied in clinical trials in combination with weekly paclitaxel administration [252,253] or an antiangiogenic drug [247]. Furthermore, many other possible metronomic-based therapies are being studied. In this regard, promising results have been attained upon combining metronomic therapy with an NC drug formulation [227,254] in melanoma [255], and colorectal [254], prostate [256], and ovarian cancer [257], thereby demonstrating how these two strategies can synergistically improve cancer treatment. To achieve metronomic dosing for ovarian cancer therapy, Amoozgar et al. have developed bilayer coated NCs to both extend paclitaxel release and improve the ability of NCs to hide from the immune system, thereby enhancing their blood circulation half-life. A comparison of encapsulated and free paclitaxel treatments demonstrated increased survival times without presenting any detectable negative effects [257]. Many therapeutic strategies based on the systemic administration of NCs, and their interaction with the tumor microenvironment, have been reported, as the nanoscale size of NCs allows them to be used for passive targeting by exploiting abnormalities in vascularity. Indeed, NCs with an appropriate size can undergo extravasation to tumor blood capillaries that present an aberrant vascular architecture and be retained in the tumor tissues due to the vascular enhanced permeability and retention (EPR) effect [258]. A significant increase (up to 100-fold) in the amount of NCs in the tumor microenvironment compared to free drugs has been reported, although it is unclear whether this is attributable solely to passive transport or whether a small percentage of NCs accumulated in the tumor stroma is delivered to the solid tumor [259,260,261]. Moreover, passive targeting is not particularly selective due to the heterogeneity of the size of the fissures in the tumor vasculature [262].

**Figure 2:**
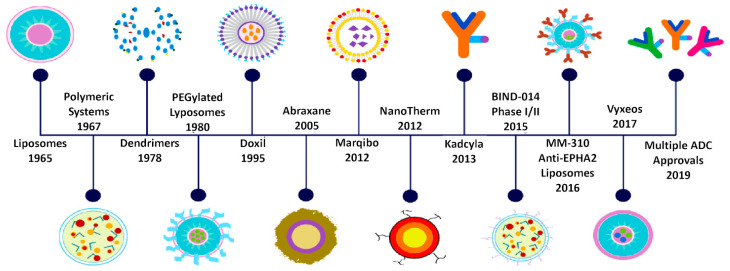
Schematic representation of the evolution of NCs towards targeted nano carriers (TNCs) in clinical cancer treatments. Some NCs formulations approved or in clinical trials, based in lipid (Doxil, Marqibo, MM-310; Vyxeos), inorganic (AMAG, NanoTherm), natural molecules (ABI-009, Abraxane), and polymers (BIND-014; AZD2811), as well as engineering monoclonal antibody for target treatment (SGT-53, Kadcyla: Traszumab conjugate with emtansine). Reprinted with permission from Ref. [275].

The biomaterials employed to produce NCs are essentially inorganic [263], lipid-based [264,265], or polymeric [266,267,268] in nature (Figure 2). All the biomaterials selected present numerous benefits as carriers for cancer therapy, especially bioavailability, biocompatibility, an ability to self-assemble, and, due to recent advancements in molecular engineering, the ability to be customized to improve their biological characteristics [269,270]. Indeed, biofunctionalized carriers facilitate molecular transport by overcoming biological barriers [271,272,273] and regulate the distribution of the drug, directing it towards the therapeutic goal, usually a selected organ, tissue, or even cell type [269,274].

The specificity of cancer therapy has been improved by using stimuli-responsive devices that achieve a therapeutic effect only in the organs affected by the tumor by directing cancer-related biomolecules to them [276,277]. Thus, NCs decorated with tumor stroma/cell targets as tumor-homing ligands facilitate the accumulation and internalization of nano-devices after interaction with certain components overexpressed in the cancer tissue, subsequently releasing anticancer drugs into solid tumors [269].

**Figure 3:**
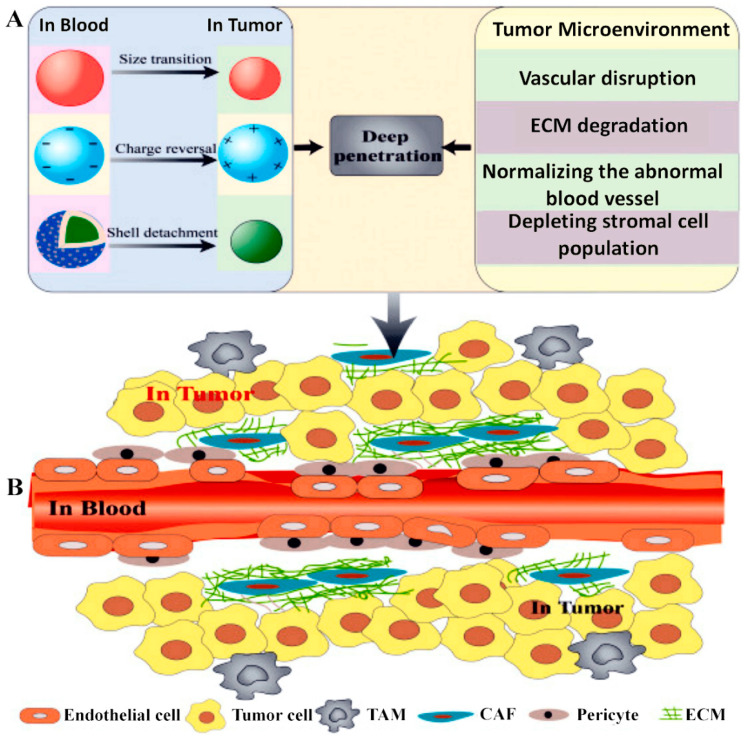
(**A**) Representation summarizing current strategies to improve the tumor penetration of NCs in response of tumor microenvironment. (**B**) Scheme of tumor vasculature and microenvironment. Reprinted with permission from Ref. [272], Copyright 2021, Elsevier.

Despite the identification of a wide set of cancer-related biomarkers [276,278,279], most of the selective therapies developed to date have proved unsatisfactory in in vivo assays due to the drug resistance observed, related to the tumor microenvironment [24,280]. Accordingly, in light of the above, tumor progression results in a stroma remodeling that deeply influences angiogenesis, invasion, and metastasis [277,281,282,283]. The tumor differs from the normal tissue microenvironment in terms of accessory, vascular, stromal, and immune cells, but also with regard to enzymes [284], pH [285], hypoxia [286], nitric oxide concentration [287], and proportion of matrix components [288]. All these physiological modifications can be used to trigger a selective response in active targeting strategies and theragnostics (Figure 3) [276,288,289,290,291].

One of the primary causes of tumor chemoresistance was identified as being related to the high interstitial pressure and hypoxic nature of tumor tissues [292]. As such, reprograming of the tumor hypoxic microenvironment is one of the main goals for overcoming drug resistance in tumor cells. For the treatment of hepatocellular carcinoma, Zan and colleagues described the results obtained using a multifunctional delivery system in which polymeric NCs were co-loaded with two natural anti-cancer molecules that are able to reduce the tumor hypoxic microenvironment decorated with a cancer-related biomarker that also increases cellular uptake. The resulting multi-functional NC significantly reduced hypoxia and tumor drug resistance, thereby increasing the therapeutic effects [293]. Although NCs directed at cancer tissues limit the possible toxicity of the therapy, optimization of the dose and administration reduces the risk of their non-specific accumulation in normal organs.

## 5. Conclusions

MC, which is preferential in a combination-therapy context, has demonstrated anti-metastatic effects in preclinical and clinical settings. The multi-target mechanism of action of MC has a potential normalizing effect on the vasculature and the tumor stroma, which is accompanied by a decrease in hypoxia, in the number of CSCs, a reduction in immunosuppression, and the promotion of an immunostimulatory microenvironment. All the above could limit tumor- and host-mediated effects in response to therapeutic strategies that generate marked stress in the TME, thus leading to therapy resistance, re-growth, and tumor progression. As such, a lack of, or limited, host-response effects to MC could maintain a stable disease state. Depending on the tumor type and stage of tumor development, the appropriate selection of drug combinations, schedules, and delivery mechanisms, such as nanocarrier-formulated chemotherapy, could improve and maintain their therapeutic efficacy.

## Figures and Tables

**Table 1:** Summaries of selected preclinical and clinical studies indicating biological mechanism of action of metronomic chemotherapy.

Cancer Type	Therapeutic Agents	In Vivo Studies	Mechanisms	Cites
Section: Metronomic Chemotherapy to Target Endothelial, Cancer, and Cancer Stem Cells.				
Pancreatic	^MC^ gemcitabine	^PC^ patient-derived xenograft	↓hypoxia ↓necrosis	[91]
Glioma	^MC^ CTX ^1^	^PC^ xenograft	↓CSCs	[74]
Pancreatic	^MC^ gemcitabine	^PC^ xenograft	↓CSCs	[92]
Section: Metronomic Chemotherapy Has a Systemic Antiangiogenic Effect by Reducing Mobi-lization and Viability of Bone-Marrow-Derived CEPs.				
Acute lymphoid leukemia (ALL)	ALL maintenance therapy (^MC^ Mercaptopurin + ^MC^ MTX ^2^)	clinical	↓blood CEPs ↑blood TSP-1	[113]
Advanced breast carcinoma	^MC^CTX/^MC^MTX +/− thalidomide	clinical	↑apoptotic blood CECs	[114]
Chronic myeloid leukemia	lenalidomide + ^MC^melphalan	clinical	↑apoptotic blood CECs	[115]
Advanced NSCLC	^MC^vinorelbine + sorafenib	clinical	dynamic changes of CECs	[116]
Lymphoma	^MC^CTX	^PC^ xenograft	↓blood CEPs	[117]
Lewis lung carcinoma (LLC)	endostar + ^MC^vinorelbine	^PC^ syngeneic mouse model	↓blood CEPs ↓microvessel density, ↓VEGF, HIF-1α	[124]
Neuroblastoma, osteosarcoma, rhabdomyo-sarcoma	^MC^topotecan + pazopanib	^PC^ xenograft	↓viable blood CEPs and CECs ↓microvessel density	[127]
Colon adenocarcinoma and liver metastasis	^MC^ capecitabine ^MC^ capecitabine + ^MC^ CTX	^PC^ xenograft	↓HIF-1α ↓intrametastatic hypoxia	[128]
Section: Metronomic Chemotherapy as Immune Modulator by Targeting Bone-Marrow-Derived Myeloid Cells.				
Pancreatic adenocarcinoma	^MTD^ gemcitabine + ^MC^ gemcitabine	^PC^ syngeneic mouse model ^PC^ xenograft	↓tumor MDSCs	[156]
Brain	^MC^ CTX (Q6day cycle)	^PC^ xenograft	↓CD11b^+^GR1^+^ bone marrow & spleen	[158,159]
Glioblastoma	^MC^ 5-FU ^3^	^PC^ xenograft	↓blood MDSCs ↑CD8^+^-T cells ↓Treg	[135]
Breast carcinoma	^MC^ capecitabine	^PC^ patient-derived xenograft	↓blood MDSCs ↑cytotoxic T cells ↓Treg	[160]
Recurrent glioblastoma	^MC^ capecitabine	clinical	↓blood MDSCs ↑CD8^+^-T cells ↑NK cells	[161,162]
Lewis lung carcinoma (LLC)	^MC^ gemcitabine + anti-Ang-2 + anti VEGF	^PC^ syngeneic mouse model	⊗MDSC recruitment in metastasis. ⊗infiltration of MΦ at metastasis site	[163]
Glioma	^MC^ temozolomide	^PC^ syngeneic rat model	↓Treg/CD4^+^-T cells in spleen	[169]
ER ^4^-positive breast	letrozol letrozol +^MC^ CTX	clinical	↓Treg	[170]
ER-positive breast	letrozol letrozol + ^MC^ vinorelbine	clinical	↑tumor infiltrating lymphocytes	[171]
Metastatic solid tumors	^MC^ CTX	clinical	↓blood Treg enhancing T and NK cell functions	[172]
Metastatic colorectal cancer	^MC^ CTX	clinical	↓blood Treg ↑CD8^+^-T cells	[173]
Section: Metronomic Chemotherapy Prevents the Pro-Stemness Function of CAFs				
Breast and pancreatic ductal adenocarcinoma	^MC^ doxorubicin ^MC^ paclitaxel ^MC^ CTX	^PC^ xenograft and human breast cancer tissues	↓ ELR^+^-chemokines CAF expression ↓expansion of CSCs	[210]

^MC^ metronomic chemotherapy, ^PC^ preclinical, ^MTD^ maximun tolerated dose, ^1^ cyclophosphamide, ^2^ methotrexate, ^3^ fluorouracil, ^4^ estrogen receptor, ↑ increase, ↓ decrease, ⊗ blocking.
